# Atrioventricular conduction in PM recipients after transcatheter aortic valve implantation: Implications using Wenckebach point measurement

**DOI:** 10.3389/fcvm.2022.904828

**Published:** 2022-07-22

**Authors:** Gemma Pelargonio, Roberto Scacciavillani, Luca Donisi, Maria Lucia Narducci, Cristina Aurigemma, Gaetano Pinnacchio, Gianluigi Bencardino, Francesco Perna, Francesco Raffaele Spera, Gianluca Comerci, Eleonora Ruscio, Enrico Romagnoli, Filippo Crea, Francesco Burzotta, Carlo Trani

**Affiliations:** ^1^Department of Cardiovascular Sciences, Fondazione Policlinico Universitario Agostino Gemelli IRCCS, Rome, Italy; ^2^Cardiology Institute, Catholic University of Sacred Heart, Rome, Italy

**Keywords:** Wenckebach point, personalized medicine, pacing, transcatheter aortic valve implantation (TAVI), atrioventricular conduction

## Abstract

**Background:**

Atrioventricular (AV) conduction disturbances requiring permanent pacemaker implantation (PPI) are a common complication after transcatheter aortic valve implantation (TAVI). However, a significant proportion of patients might recover AV conduction at follow-up.

**Objectives:**

The aim of our study was to evaluate the recovery of AV conduction by determination through Wenckebach point in patients with PPI and therefore identify patients who could benefit from device reprogramming to avoid unnecessary RV pacing.

**Methods:**

We enrolled 43 patients that underwent PM implantation after TAVI at our Department from January 2018 to January 2021. PM interrogation was performed at follow-up and patients with native spontaneous rhythm were further assessed for AV conduction through WP determination.

**Results:**

A total of 43 patients requiring a PM represented the final study population, divided in patients with severely impaired AV conduction (no spontaneous valid rhythm or WP < 100; 26) and patients with valid AV conduction (WP ≥ 100; 17). In the first group patients had a significantly higher number of intraprocedural atrioventricular block (AVB) (20 vs. 1, *p* < 0.005), showed a significant higher implantation depth in LVOT (7.7 ± 2.2 vs. 4.4 ± 1.1, *p* < 0.05) and lower ΔMSID (−0.28 ± 3 vs. −3.94 ± 2, *p* < 0.05).

**Conclusion:**

AV conduction may recover in a significant proportion of patients. In our study, valve implantation depth in the LVOT and intraprocedural AV block are associated with severely impaired AV conduction. Regular PM interrogation and reprogramming are required to avoid unnecessary permanent right ventricular stimulation in patients with AV conduction recovery.

## Graphical Abstract

Study design and main results are illustrated. TAVI, Transcatheter Aortic Valve Implantation; PM, pacemaker; WP, Wenckebach point; AVB, atrioventricular block; LBBB, left bundle branch block; LBB, left bundle branch; RBB, right bundle branch; ID, implantation depth; HisB, His bundle; HAVB, high-degree atrioventricular block; LVOT ID, left ventricular outflow tract implantation depth; DMSID, difference membranous septum-implantation depth. 
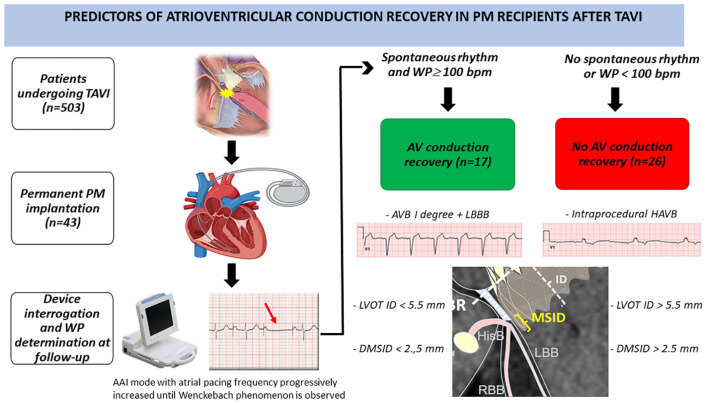

## What’S New

- Conduction disturbances after TAVI are a frequent complication of the procedure but selection of patients more likely to need permanent pacemaker implantation and benefit from it remains challenging.
- We assessed atrioventricular conduction recovery through Wenckebach point determination in patients who received a pacemaker after TAVI.
- We sought to identify predictors of AV conduction recovery and to guide pacing strategies, aiming to minimize right ventricular pacing in subjects who recovered AV conduction.
- Wenckebach point determination could be a more reliable tool to assess AV node function after TAVI, compared to percentage of pacing and spontaneous rhythm assessment.

## Introduction

Transcatheter Aortic Valve Implantation (TAVI) is emerging as a safe, less invasive, and faster procedure in patients with severe aortic stenosis compared to Surgical Aortic Valve Replacement (SAVR), even in subjects at low surgical risk (1–3). However, it still pays the burden of a higher risk of conduction abnormalities and consecutive permanent pacemaker implantation (PPI) due to the anatomical proximity of the atrioventricular (AV) conduction system to the area of the implanted prosthetic valve (4). The TAVI procedure implies a series of technical steps (left ventricle catheterization, stiff wire allocation, balloon valvuloplasty) that may cause transient mechanical interaction with the AV conduction system. Theoretically, a proportion of patients judged to require PPI after TAVI, have the potential to recover AV conduction. Better understanding of this phenomenon and eventual recognition of these patients might avoid unnecessary PPI after TAVI with consequent morbidity and mortality (5).

The aim of our study is to evaluate the recovery of the AV conduction through Wenckebach point (WP) determination in patients undergoing PPI after TAVI.

We have collected clinical, anatomical, and procedural parameters of patients who recovered native AV conduction at follow up, in order to assess possible predictors of AV conduction recovery after TAVI (central illustration shows study design and main results).

## Materials and methods

### Transcatheter aortic valve implantation program and procedure

The management of patients with aortic stenosis in our center is routinely performed according to internal guidelines (in the institutional clinical pathway dedicated to patients with heart valve diseases).^1^ Briefly, each patient underwent a thorough clinical and echocardiographic evaluation before the procedure according to the standard practice of our Center all patients and were referred for TAVI on the bases of formal, multidisciplinary, Heart Team discussion. Clinical data and procedure details were prospectively entered into a dedicated database that allowed previously to assess the impact of EuroSCORE on coronary interventions (6) and the safety of trans-radial procedures (7). Patients’ surgical risk was graded according to the Society of Thoracic Surgeons (STS) predicted operative mortality at the time of Heart Team consultation. TAVI risk was graded according to the STS/American College of Cardiology Transcatheter Valve Therapy (8). In-hospital clinical outcomes were prospectively recorded since the continuous monitoring of in-hospital clinical outcomes for TAVI is part of our institutional clinical pathway dedicated to patients with heart valve diseases.

TAVI was indicated by the institutional Heart Team for the treatment of severe symptomatic aortic stenosis. All TAVI were conducted according to a procedural plan performed on the basis of computed tomography (CT) scan. For each patient, the CT scan was revised by at least two operators to assess the potential suitability for TAVI implantation. Transfemoral approach was considered the preferred option, other accesses being considered in the case of absence of suitable aorto-iliac-femoral anatomy due to insufficient lumen size, extreme tortuosity and/or severe atherothrombosis. TAVI was performed following the manufacturer’s best practice recommendations. Transfemoral TAVI was conducted according to our previously reported technique (7). Both self-expanding and balloon-expandable prostheses were used. Balloon aortic valvuloplasty was performed when deemed necessary by the operator.

Left ventricular outflow tract (LVOT) implantation depth (ID) was measured as the distance between the lower end of the transcatheter heart valve frame and the lowest part of the non-coronary and left cusp, as previously described (9, 10).

All patients signed a dedicated informed consent to the study procedure, which included the authorization to database insertion and clinical follow-up assessment. The study was approved by the Institutional Committee on Human Research at our Institution.

### Study population and baseline clinical-radiological data

**FIGURE 1:**
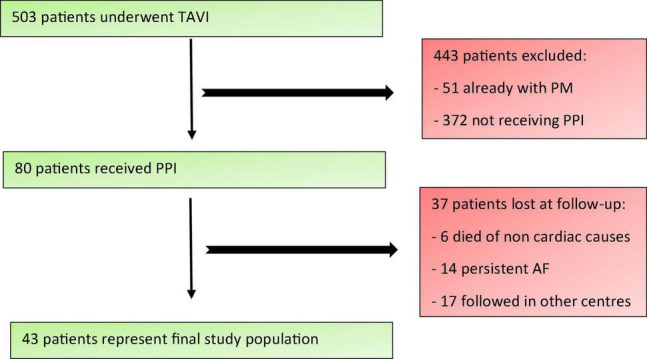
Study population enrolment criteria. TAVI, Transcatheter Aortic Valve Implantation; PM, pacemaker; PPI, permanent pacemaker implantation; AF, atrial fibrillation.

A total of 503 patients underwent TAVI at our Department from January 2018 to January 2021. Patients who had a pre-existing pacemaker were excluded from the analysis. Of the 80 patients (15.9%) who received PPI, 37 were lost to follow-up: 6 patients (1.2%) died from non-cardiovascular causes, 14 had permanent atrial fibrillation (2.8%) and 17 were followed in other centers distant from ours (3.4%). A total of 43 patients represented the final study population (Figure 1). Clinical, echocardiographic, anatomical, CT scan and procedural characteristics (both TAVI and PM implantation procedures) were collected.

Membranous septum was measured in a dedicated CT coronal view as the perpendicular distance from the annular plane to the beginning of the muscular septum and then it was calculated the difference between the LVOT ID under the non-coronary cusp and membranous septum (DMSID), as described elsewhere (9).

### Indications for permanent pacemaker implantation

Eighty patients received PPI due to new continuous high-degree AV block (HAVB) (20, 47%), left bundle branch clock (LBBB) + AVB I grade (10, 23%), paroxysmal complete AVB (7, 16%), AVB II grade Mobitz 2 (4, 9%), AVB 2:1 (2, 5%), in accordance with international guidelines and consensus documents (11–14). The protocol followed by the center was to wait 24/48 before implanting a PM if the block developed during the procedure, while a longer period of observation was waited whenever other rhythm or conduction disturbances were observed after the procedure, and a PM eventually was implanted based on ECG and symptoms’ features.

All patients were discharged with PM programming according to current guidelines (11, 12).

### Follow up after permanent pacemaker implantation and atrioventricular node function assessment through Wenckebach point determination

We followed 43 patients in our hospital based on our standard care of patients who receive a pacemaker (PM) and analyzed AV node function through PM device interrogation at follow up visits. Clinical and device follow-up were done at least 3 months after implantation up to 25 months. Median follow-up was 13 months.

**FIGURE 2:**
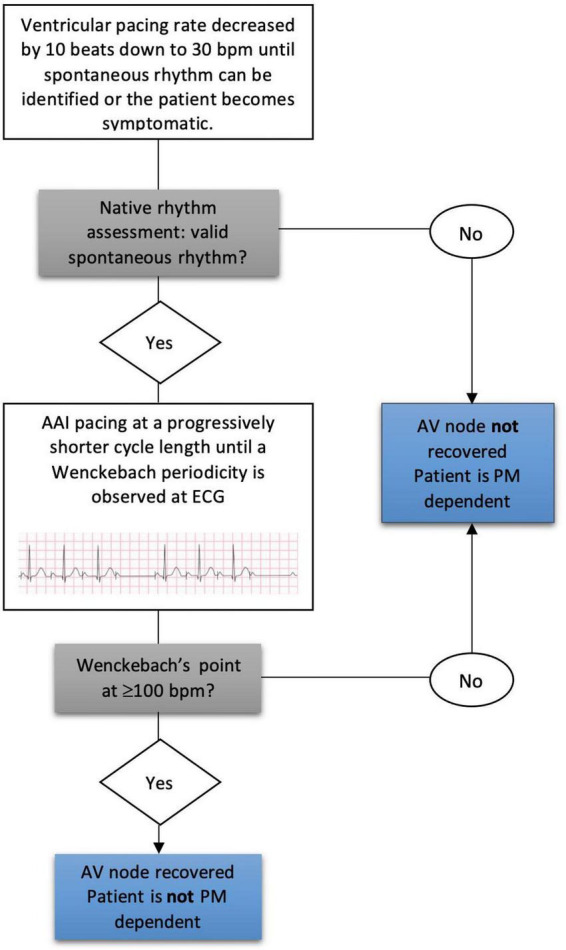
Wenckebach point determination at follow-up and pacemaker dependency assessment. AV, atrioventricular; PM, pacemaker.

To evaluate AV node conduction PM pacing was temporarily decreased by 10 beats down to 30 bpm to evaluate spontaneous conduction rhythm. Subjects with a ventricular escape rate of less than 30 beats/min on device interrogation were considered PM dependent (PMD) as previously described (15, 16). In patients who presented a spontaneous rhythm > 30 bpm we analyzed AV node function through AAI pacing during ECG monitoring. We paced the right atrium at a progressively shorter cycle length to assess AV node conduction and thus determine anterograde WP. WP was defined as the highest atrial pacing rate at which AVB was observed for the first time, in the form of Wenckebach periodicity. Based on the value of heart rate at which WP occurred, patients were defined as having valid spontaneous rhythm if their WP was ≥ 100 bpm (cycle length ≤ 600 ms), whereas patients with a WP < 100 were considered with severely impaired AV conduction (Figure 2). For convenience, valid AV conduction patients were defined those who had a preserved AVN function with a WP ≥ 100 bpm, while severely impaired AV conduction patients those with a WP less than 100 bpm or a spontaneous rhythm of less than 30 bpm or a complete AV block. The cut-off value for WP determination was chosen in accordance with previously published literature and considered appropriate for this elderly population by the authors (17).

### Statistical analysis

Continuous variables are expressed as mean ± standard deviation or median ± interquartile ranges for non-normally distribute variables. Categorical differences between groups were examined using Pearson’s chi-square test, while unpaired Student’s *t*-test or the Mann-Whitney U test for non-normally distributed variables were used to compare differences between means. Cox proportional hazards regression analysis was performed to assess the association of baseline clinical variables with recovery of AV conduction. The results of such analysis are presented as odds ratios (ORs) and 95% confidence interval (CI). A *P*-value < 0.05 was considered significant for all statistical determination. All analyses were performed using SPSS Statistics 23 software for Windows.

## Results

Baseline clinical characteristics of the study population are summarized in Table 1. There were no significant differences between clinical, electrocardiographic, and echocardiographic parameters in the two groups. Twenty-one (49%) patients had a previous diagnosis of ischemic cardiac disease. A preexisting bundle branch block was noted in 8 (19%) patients, 3 (7%) of them showed a left bundle block and 5 (12%) a right bundle block (Table 1). Forty-one patients (95%) underwent transfemoral aortic valve implantations. Mean follow-up after TAVI were 21 ± 13 months in the severely impaired AV conduction group and 14 ± 11 months in the valid AV conduction group. After follow-up visit and PM interrogation, 26 (60%) patients did not show native spontaneous rhythm > 30 bpm (HAVB or CAVB) or had WP < 100 bpm and thus were defined with severely impaired AV conduction. Of these, 6 (14%) had a spontaneous native rhythm but when investigated showed a WP < 100 and were then considered with severely impaired AV conduction. 17 patients (40%) had native spontaneous rhythm and showed a WP ≥ 100 bpm and thus were considered with valid AV conduction.

**TABLE 1:** Clinical, ECG and echocardiographic characteristics.

	Severely impaired AV conduction (WP < 100 or not detected) *N* = 26	Valid AV conduction (WP ≥ 100) *N* = 17	*P*-value
**Patient clinical characteristics**			
Age	79 ± 11	80 ± 8	0.75
Males	17 (65%)	10 (59%)	0.66
Diabetes mellitus	8 (31%)	8 (47%)	0.28
Cigarette smoking	3 (12%)	1 (6%)	0.53
Hypercholesteremia	13 (50%)	9 (53%)	0.85
Arterial hypertension	23 (88.5%)	14 (82.4%)	0.57
Obesity	8 (30.8%)	5 (29%)	0.92
Respiratory insufficiency	6 (60%)	4 (40%)	0.97
Peripheral artery disease	3 (12%)	4 (24%)	0.29
Renal insufficiency (eGFR < 30 ml/min)	8 (30.8%)	5 (29%)	0.92
NYHA class III-IV	9 (35%)	4 (26%)	0.44
Previous heart surgery	5 (19%)	2 (12%)	0.52
Coronary artery disease	9 (35%)	6 (35%)	0.97
Paroxysmal AF	5 (17%)	1 (6%)	0.22
**ECG features pre TAVI**			
HR pre TAVI (bpm)	61 ± 20	65 ± 12	0.29
PR pre TAVI (ms)	172 ± 210	200 ± 50	0.09
QRS pre TAVI (ms)	114 ± 52	109 ± 36	0.47
QT pre TAVI (ms)	425 ± 58	401 ± 60	0.28
RBBB pre TAVI	5 (21%)	2 (12%)	0.52
LBBB pre TAVI	3 (13%)	2 (12%)	0.98
**Echocardiographic features**			
LVEF%	55 ± 12	54 ± 12	0.80
Max aortic gradient pre (mmHg)	63 ± 23	64 ± 29	0.90
Mean aortic gradient pre (mmHg)	48 ± 13	52 ± 8	0.27
AVA (cm^2^)	0.77 ± 0.11	0.80 ± 0.11	0.99

*Values expressed as mean ± standard deviation or percentages.*

*PM, pacemaker; WP, Wenckebach point; BMI, body mass index; Egfr, estimated glomerular filtration rate; NYHA, New York Heart Association; AF, atrial fibrillation; TAVI, Transcatheter Aortic Valve Implantation; HR, heart rhythm; RBBB, right bundle branch block; LBBB, left bundle branch block; LVEF, left ventricular ejection fraction; AVA, aortic valve area.*

Pacemakers were all programmed in DDD modality, with a lower rate of 60 bpm in 88% of cases (*n* = 38), rate responsiveness active in only 9% of them (*n* = 4) and minimum ventricular pacing algorithms enabled in 16% of subjects (*n* = 7).

**FIGURE 3:**
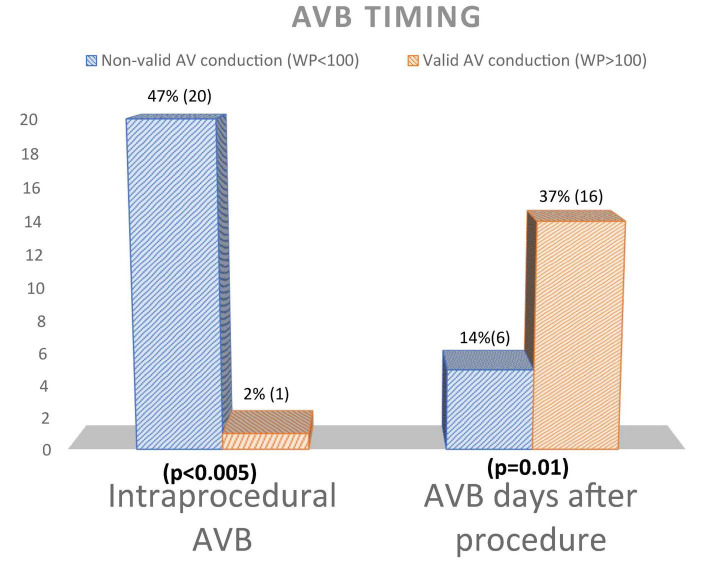
Atrioventricular block timing differences between patients with and without AV conduction recovery. AV, atrioventricular; WP, Wenckebac point; AVB, atrioventricular block.

Patients with severely impaired AV conduction had a significantly higher number of intraprocedural AVB (20 vs. 1, *p*-value < 0.005), while in the other group patients displayed AVB several days after the procedure (2 ± 4 vs. 6 ± 3, *p*-value 0.01) (Figure 3).

Particularly, in the severely impaired AV conduction group patients implanted a PM earlier after TAVI (2 ± 4 days vs. 6 ± 3 days respectively, *p* = 0.001), with consequent higher rate of post-procedural temporary PM (18 vs. 5, *p*-value 0.012) (Table 2).

**TABLE 2:** Anatomical, procedural and follow-up characteristics.

	Severely impaired AV conduction (WP < 100 or detected, *n* = 26)	Valid AV conduction (PW ≥ 100, *n* = 17)	*P*-value
**Anatomical features at CT**			
Membranous septum length (mm)	8.2 ± 3.5	8.0 ± 2.5	0.80
LVOT diameter/aortic annulus	22 ± 4	23 ± 4	0.43
Bicuspid aorta	4 (15%)	0 (0%)	0.12
**Procedural characteristics**			
STS mortality	3.3 ± 1.9	2.8 ± 1.6	0.16
Valve index (valve size/LVOT × 100)	1.33 ± 0.17	1.26 ± 0.21	0.24
LVOT ID under non-coronary cusp (mm)	7.7 ± 2.2	4.4 ± 1.1	**0.0001**
LVOT ID under left coronary cusp (mm)	7.08 ± 2.78	8.41 ± 3.39	0.26
DMSID (mm)	−0.28 ± 3	−3.94 ± 2	**0.0001**
TAVI transfemoral	25 (96%)	16 (94%)	0.76
Pre-dilatation	21 (81%)	14 (82%)	0.90
Post-dilatation	5 (19%)	3 (18%)	0.90
Self-expandable	25 (96%)	14 (82%)	0.13
EVOLUT PRO	15 (58%)	8 (47%)	0,355
EVOLUT R	11 (42%)	4 (24%)	0,175
CORE VALVE	1 (2%)	0 (0%)	0,395
SAPIEN3	0 (0%)	3 (18%)	0,06
PORTICO	0 (0%)	1 (2%)	0,395
Temporary PM	18 (69%)	5 (29%)	**0.01**
**ECG features post TAVI**			
HR post TAVI (bpm)	66 ± 15	75 ± 23	0.06
QRS post TAVI (ms)	160 ± 59	138 ± 35	**0.016**
QT post TAVI (ms)	447 ± 68	402 ± 48	**0.002**
RBBB post TAVI	5 (21%)	4 (24%)	0.66
LBBB post TAVI	7 (27%)	7 (41%)	0.33
AF at follow-up	5 (19%)	5 (30%)	0.44
**AV block features**			
AV block in the days after the procedure	5 (19%)	14 (82%)	**<0.0001**
Intraprocedural HAVB	20 (77%)	1 (6%)	**<0.0001**
AVB I + LBBB	2 (8%)	8 (48%)	**0.003**
AVB II 2:1	0 (0%)	2 (12%)	0.15
AVB II Mobitz 2	1 (4%)	3 (18%)	0.13
Advanced AVB	3 (12%)	1 (6%)	0.53
Paroxysmal AVB III	2 (8%)	5 (29%)	0.06
Complete AVB III	19 (73%)	1 (6%)	**<0.0001**
Days to implantation from TAVI	2 ± 4	6 ± 3	**0.001**
Follow-up months from PM implantation	21 ± 13	13 ± 11	0.07

*Values expressed as mean ± standard deviation or percentages.*

*PM, pacemaker; WP, Wenckebach point; CT, computed tomography; LVOT, left ventricular outflow tract; STS, Society of Thoracic Surgeons; ID, implantation depth; DMSID, difference membranous septum-implantation depth; TAVI, Transcatheter Aortic Valve Implantation; HR, heart rhythm; RBBB, right bundle branch block; LBBB, left bundle branch block; AF, atrial fibrillation; AV, atrioventricular; HAVB, high grade atrioventricular block.*

*Bold values are significant P values.*

**FIGURE 4:**
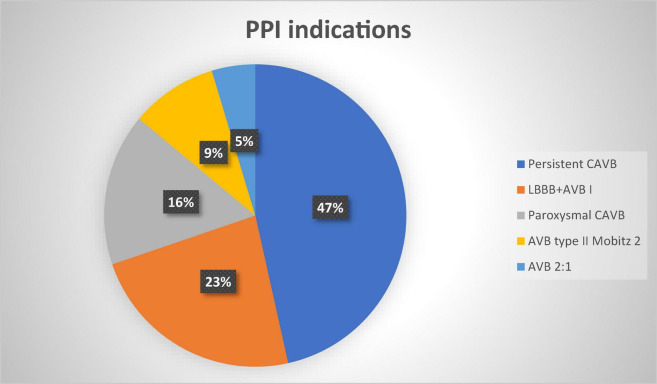
Permanent pacemaker indications percentages. PPI, permanent pacemaker implantation; CAVB, complete atrioventricular block; LBBB, left bundle branch block; AVB, atrioventricular block.

The indications to PPI were as follows: persistent complete AVB/HAVB (20 patients, 47%); new onset LBBB + AVB I degree (10 patients, 23%), paroxysmal complete AVB (7 patients, 16%); AVB type II Mobitz 2 (4 patients, 10%); AVB with 2:1 conduction (2 patients, 5%) (Figure 4).

Particularly the most frequent indication for PPI was complete AVB in severely impaired AV conduction patients and LBBB + AVB I in valid AV conduction patients (Table 2).

Severely impaired AV conduction patients showed a significant higher LVOT ID (7.7 ± 2.2 vs. 4.4 ± 1.1, *p*-value < 0.05) and lower DMSID (−0.28 ± 3 vs. −3.94 ± 2, *p*-value < 0.05).

### Predictors of permanent pacemaker implantation

LVOT ID > 5.5 mm and DMSID > 2.5 mm were identified as possible cut-off values by means of receiver operating characteristic (ROC) curves.

At Cox regression analysis, LVOT ID, DMS ID, intraprocedural block and complete AVB were associated with no AV conduction recovery (Table 3).

**TABLE 3:** Variables associated with severely impaired AV conduction at Cox regression analysis.

	OR	95% CI	*P*-value
**Cox regression analysis**			
NYHA class III or IV	0.7	0.30–1.64	0.40
Coronary artery disease	0.63	0.27–1.48	0.29
LVOT ID	1.45	1.17–1.78	**0.001**
Intraprocedural AVB	3.56	1.33–9.56	**0.01**
Temporary PM	2.26	0.95–5.44	0.07
Complete AVB	2.99	1.19–7.53	**0.02**
DMS ID	1.12	1.05–1.37	**0.008**
AVB I + LBBB	0.38	0.09–1.62	0.19
AVB II Mobitz 2	0.57	0.08–4.21	0.58

*OR, odds ratio; CI, confidence interval; PM, pacemaker; NYHA, New York Heart Association; LVOT ID, left ventricular outflow tract implantation depth; AVB, atrioventricular block; DMSID, difference membranous septum-implantation depth. Bold values are significant P values.*

## Discussion

To the best of our knowledge this is the first study to assess AV node function and recovery in PPI after TAVI, in a follow up period beyond 1 year. AV node recovered its normal conduction in 40% of the implanted patients. WP > 100 bpm at follow up was associated with a later AVB appearance from TAVI, a higher LVOT ID and a lower DMSID.

Permanent pacing may bring some potential negative effects on patients, then assessing AV node function and the percentage of ventricular pacing may become of crucial importance. Indeed, chronic pacing after TAVI is associated with increased morbidity and mortality as showed by different studies (18–21). Moreover, major complications associated to conventional PM systems are still common and represent a significant burden to healthcare systems as they generate substantial costs (22).

### Atrioventricular conduction recovery

Many studies in the literature investigated the incidence and predictors of PM implantation with the final aim of reducing PPI rates through pre-procedural assessment (9, 23, 24). Recently, a meta-analysis involving 43 studies and encompassing 29 113 patients, reported the PPI rates to range from 6.7 to 39.2%. Independent predictors for PPI following TAVI have been widely investigated and the principal ones identified were age, previous RBBB, self-expanding valve type, and valve implantation depth (25).

It has been widely hypothesized that conduction abnormalities after TAVI are due to mechanical pressure of the implanted valve to the AV bundle, which leads to edema and inflammation in the local tissue. Although a multidisciplinary expert consensus suggested waiting up to 24 h after TAVI and more recently ESC guidelines recommended waiting for 24–48 h after TAVI to confirm the indication to permanent pacemaker implantation (13, 26), local damage may resolve several days after implantation and lead to conduction recovery after implantation.

Few studies have investigated the rates and predictors of pacemaker dependency after TAVI and none have used methods to investigate AV node recovery to detect patients who are potentially not pacing-dependent, as we did through the identification of the WP at follow-up.

WP determination measures AVN conduction but may help unmask potential damages below AVN and in the His-Purkinje system that are not visible at normal values of heart rates. There is no certainty of the site of block in most TAVI, probably mainly located in the area of the membranous septum, where the His system generally lyes, but they may be more proximal or distal to this point. Recovery of conduction might be easily attributable to a higher location of the block, closer to the AVN, rather than the His or below the His. WP assessment may help differentiating AVN conduction and a potential recovery along the AV electrical system. Whenever the ventricle is able to conduct all the pacing beats over 100 bpm, the chance to assume recovery conduction below the AVN becomes more reliable.

Most studies defined PM dependency if ventricular pacing (VP) was > 90% (or PM non-dependency if VP < 5%) (23, 27), with the presence of a spontaneous rhythm with PM stimulation progressively decreased at 30 or 40 bpm at device interrogation (16) or with their combination (9).

However, the percentage of VP does not give realistic information on the AV conduction. VP may be influenced by different factors such as increased pacing during the night or modality of pacing which are those “out of the box,” based on standardized algorithms rather than tailored on the patient. In addition to that, having a spontaneous rhythm faster than 30 or 40 bpm does not mean that this is a valid rhythm for daily activities.

For example, 56% (24) of our patients could be considered potential non-pacing dependent with these investigation criteria (ventricular pacing less than 5% and presence of native rhythm with VVI 30 stimulation), but just 40% (17) of our patients could be considered with valid AV conduction according to the WP being above 100 bpm and thus with proper AV node conduction recovered. This highlights the concept that definitions of pacemaker dependency used until now carry intrinsic pitfalls and should be used with caution.

Interestingly, in the group of patients with recovered AV conduction (WP > 100 bpm), the pacemaker, as it was set, indicated a ventricular pacing equal or higher than 5% in 11 out of 17 patients (65%). This implies that a significant proportion of patients who recovered AV conduction is programmed with potentially avoidable chronic right ventricular pacing. The evaluation of WP in patients who show spontaneous native rhythm at follow-up may give more detailed information about AV node conduction after TAVI and may help to identify non-pacemaker dependent patients who could benefit from PM reprogramming.

Our results suggest therefore that in patients with recovered AV conduction, AAI-DDD modality should be encouraged since it promotes minimal ventricular pacing (MinVP) and thus a more physiological and synchronous ventricular contraction. Indeed, patients of this study who exhibited valid AV conduction were reprogrammed with algorithms that avoid unnecessary RV pacing, that are available on modern devices.

This concept is in accordance with findings from recent studies that reported that patients programmed with conventional DDD after TAVI (21) or who needed PPI after TAVI (5), had significantly higher morbidity and mortality. In another study, chronic right ventricular pacing in patients implanted after TAVI was associated with significantly higher rates of adverse clinical events (including mortality) and with significantly lower LVEF at 1 year. On top of that, unnecessary chronic ventricular pacing suppresses spontaneous native rhythm and leads to early battery depletion with the associated costs of generator replacement and its inherent risks (pocket infection, hematoma…) (22).

### Predictors of atrioventricular conduction recovery

LVOT ID of prosthesis and membranous septum (MS) length have been associated with PPI and no AV conduction recovery. Due to the anatomical course of the AV bundle along the lower border of the MS (which lies close to the aortic annulus) in most subjects, the frame of a low-implanted TAVI might permanently injure the branches emerging on the endocardial surface between the MS and the muscular ventricular septum. Thus, in subjects with a short MS, the AV bundle is more likely to be compressed by the prosthesis, even if optimal implantation depth is achieved. Previously, Gaede et al. (23) have found the only predictors of a lack of recovery of the AVB to be prior RBBB, higher mean aortic valve gradients and post-dilatation of the prosthesis. More recently, Nai Fovino et al. (9) reported that baseline electrocardiographic characteristics, MS length, implantation depth, and type of implanted TAVI were not predictive of long-term pacemaker dependency, while a difference between implantation depth in the LVOT and membranous septum length accounting for ≥ 3 mm and the presence of LVOT calcification under the LCC were the only independent predictors of 30-day pacemaker dependency after TAVR.

In our study, depth in LVOT under NCC and DMS ID, were associated with severely impaired AV conduction, remarking the anatomical importance of valve implantation depth in relation to AV bundle anatomical position. Notably depth in LVOT under LCC did not differ between groups and this finding, anatomically, is in accordance with the location of the His bundle that runs in between the right and non-coronary cusps (28). Indeed, also the difference between depth in LVOT and membranous septum length (DMSID) was significantly different in the two groups. This finding may suggest that reducing the valve implantation depth intra-procedurally, before the definite landing of the valve, might lead to better AV conduction recovery and less PM dependency after TAVI, other than decreased risk of PPI. This comes in accordance with a recent study from Sammour et al. (29) demonstrating that higher implantation in the LVOT results in significant reduction in conduction abnormalities and permanent pacemaker requirement without compromising procedural safety or valve hemodynamics. Of course, the risk of pacemaker dependency needs to be counterbalanced with the drawbacks of a high valve implantation, such as the risk of valve embolization and coronary access impairment, particularly in patients with a short MS (30).

Contrary to other data in literature (31), in our study we did not find a difference between the types of valves employed, especially between self-expanding and balloon-expanding ones, probably because of the relatively small sample size and the prevalent use of self-expanding prosthetic valves.

We also investigated electrocardiographic patterns of procedural conduction abnormalities and found that intraprocedural block, mainly third degree complete AVB, was strongly associated to no AV conduction recovery.

This finding illustrates that AV conduction hardly recovers in patients who develop AVB during TAVI procedure and thus patients showing this characteristic may undergo direct implantation minimizing hospital stay and costs. This phenomenon may be explained by the occurrence of direct and irreversible mechanical damage to the conduction system in this situation; instead, AVB that occurs in the subsequent days is probably caused by edema and inflammation and is more likely to resolve over time. Interestingly, the most frequent indication for PPI after TAVI (LBBB + AV block 1st degree), was not associated with severely impaired AV conduction. LBBB + AVI 1st degree is a common indication of PM implantation after TAVI (13) and this finding may suggest a watchful waiting approach in these patients since chances of recovery are high. It has also to be emphasized that, on the other hand, some patients actually do recover a valid AV conduction after TAVI and PPI and in those patients every effort should be made in terms of drug therapy and device programming to favor native electrical conduction.

Finally, most studies have investigated PM dependency up to 1 year (9, 23, 24), so this is the first of this kind that extends the follow-up after 12 months and these findings might suggest that in a significant proportion of patients AV conduction persists over 1 year.

### Study limitations

The present study carries the inherent limitations of a single-center analysis and relatively small sample size, which may hamper the strength of our results, that must therefore be considered relative to a small cohort. Moreover, AV node conduction through the WP determination was not evaluated before TAVI or immediately after TAVI and thus no information is known about AV conduction at baseline. Patients were followed up 3–25 months after TAVI without prespecified fixed intervals; however, most AV conduction disturbances in post TAVI patients occur in the acute period, in the first 24 h up to 7 days and even in healthy subjects Wenckebach point may change in time due to the autonomic nervous system balance. We acknowledge that WP may change with exercise testing or isoproterenol infusion and therefore our WP determination has to be considered a basal resting measure. Having done WP testing at least 3 months after TAVI, we assumed we were in a moment of achieved stabilization of the prosthetic valve with the surrounding conduction system. We also do not have WP information in patients after TAVI without PPI; in this cohort there may be AV conduction alterations with the substantial difference that they did not meet criteria for PPI. The WP cut-off to define PM dependency was set at 100 bpm since this value was considered by the authors appropriate for this elderly and high-risk population.

## Conclusion

Our study shows that a significant proportion of patients who received pacemakers for conduction abnormalities post-TAVI have recovery of AV conduction and might be non-pacemaker-dependent over time.

Complete AV block occurring during the procedure and increased LVOT ID predict severely impaired AV conduction at follow-up. In these patients a more expedited pacemaker implantation, minimizing hospital stay, may be considered, while longer observation periods prior to pacemaker placement might be justified in patients showing I degree AVB + LBBB.

Reprogramming with algorithms to minimize ventricular pacing should be used in patients who have recovered from AV conduction dysfunction to avoid ventricular dyssynchrony and long-term complications.

## Data Availability Statement

The raw data supporting the conclusions of this article will be made available by the authors, without undue reservation.

## Ethics statement

The studies involving human participants were reviewed and approved by Policlinico Gemelli Ethics Committee. The patients/participants provided their written informed consent to participate in this study.

## Author contributions

All authors listed have made a substantial, direct, and intellectual contribution to the work, and approved it for publication.

## Conflict of Interest

The authors declare that the research was conducted in the absence of any commercial or financial relationships that could be construed as a potential conflict of interest.

## Publisher’s Note

All claims expressed in this article are solely those of the authors and do not necessarily represent those of their affiliated organizations, or those of the publisher, the editors and the reviewers. Any product that may be evaluated in this article, or claim that may be made by its manufacturer, is not guaranteed or endorsed by the publisher.

## Footnotes

1
https://www.policlinicogemelli.it/en/patient-services/care-pathways/valvulopatia/
